# Factor analysis of treatment outcomes from a UK specialist addiction service: Relationship between the Leeds Dependence Questionnaire, Social Satisfaction Questionnaire and 10-item Clinical Outcomes in Routine Evaluation

**DOI:** 10.1111/dar.12146

**Published:** 2014-05-07

**Authors:** Caroline Fairhurst, Jan R Böhnke, Rhian Gabe, Tim J Croudace, Gillian Tober, Duncan Raistrick

**Affiliations:** 1 York Trials Unit, University of YorkYork, UK; 2 Mental Health and Addiction Research Group (MHARG), Hull York Medical School and Department of Health Sciences, University of YorkYork, UK; 3 Hull York Medical School, University of YorkYork, UK; 4 Leeds Addiction UnitLeeds, UK

**Keywords:** factor analysis, substance dependence, patient-reported outcome measure, social satisfaction, psychological distress

## Abstract

**Introduction and Aims:**

To examine the relationship between three outcome measures used by a specialist addiction service (UK): the Leeds Dependence Questionnaire (LDQ), the Social Satisfaction Questionnaire (SSQ) and the 10-item Clinical Outcomes in Routine Evaluation (CORE-10).

**Design and Method:**

A clinical sample of 715 service user records was extracted from a specialist addiction service (2011) database. The LDQ (dependence), SSQ (social satisfaction) and CORE-10 (psychological distress) were routinely administered at the start of treatment and again between 3 and 12 months post-treatment. A mixed pre/post-treatment dataset of 526 service users was subjected to exploratory factor analysis. Parallel Analysis and the Hull method were used to suggest the most parsimonious factor solution.

**Results:**

Exploratory factor analysis with three factors accounted for 66.2% of the total variance but Parallel Analysis supported two factors as sufficient to account for observed correlations among items. In the two-factor solution, LDQ items and nine of the 10 CORE-10 items loaded on the first factor *>*0.41, and the SSQ items on factor 2 with loadings *>*0.63. A two dimensional summary appears sufficient and clinically meaningful.

**Discussion and Conclusions:**

Among specialist addiction service users, social satisfaction appears to be a unique construct of addiction and is not the same as variation due to psychological distress or dependence. Our interpretation of the findings is that dependence is best thought of as a specific psychological condition subsumed under the construct psychological distress. *[Fairhurst C, Böhnke JR, Gabe R, Croudace TJ, Tober G, Raistrick D. Factor analysis of treatment outcomes from a UK specialist addiction service: Relationship between the Leeds Dependence Questionnaire, Social Satisfaction Questionnaire and 10-item Clinical Outcomes in Routine Evaluation.* Drug Alcohol Rev *2014;33:643–650]*

## Introduction

Measuring the scope and severity of a complex health problem like addiction informs and guides treatment. Besides the addiction itself, patients often suffer from anxiety and depression; these complex needs often result in less optimal outcomes [1]. Well-chosen outcome measures are integral to the treatment process and can be harnessed, for example, to give feedback on progress and to reassess treatment needs. Choosing among possible measures presupposes that an appropriate number of dimensions for the outcome assessments is already established, but often practical experience or diagnostic theory is lacking or inconclusive. One influential model, the phase model of psychotherapy by Howard et al. [2], entails improvement in several domains: subjectively experienced well-being, resolution of symptoms and life problems, and enhancement of life-functioning. Appropriate service user-based outcome measurement strategies are required to evaluate these problems and measure change in people progressing through treatment for psychological disorders, without undue burden of assessment (unnecessary or duplicate measurement).

National Institute for Health and Clinical Excellence (NICE) clinical guidelines recommend a comprehensive assessment for all adults referred to specialist addiction services, evaluating multiple areas of need, including consumption, physical health and psychological distress using relevant and validated clinical tools [3]. Advised treatment goals are tailored depending on the severity of dependence, presence of psychiatric comorbidities and level of social support. These outcomes should be monitored routinely through treatment to inform the continuation of both psychological and pharmacological interventions.

Such theoretical (Howard) and normative (NICE) models of treatment justify the use of multiple outcome instruments during the treatment of psychological disorders. Common domains in the measurement of addiction include dependence, psychosocial or emotional maladjustment and severity of symptoms [4]. This study concerns three measures that have been extensively investigated: (i) the Leeds Dependence Questionnaire (LDQ), a 10-item scale measuring dependence on psychoactive substances [5]; (ii) the 10-item Clinical Outcomes in Routine Evaluation questionnaire (CORE-10) measuring psychological distress [6]; and (iii) the Social Satisfaction Questionnaire (SSQ), an eight-item instrument adapted from the Social Problems Questionnaire [7] assessing satisfaction with social circumstances [8].

These measures are designed for service user completion, are of universal application with regard to substance and populations and are widely perceived to be clinically relevant and comprehensive. Many other scales are available but few have adequate evaluation of their psychometric properties, as judged by a quality framework [9] that scores such things as the psychometric properties and user acceptability of scales. The LDQ and the SSQ have extensive and independent validation 8,10–14 and the psychometric properties of the CORE-10 have been comprehensively investigated [15]. The development of the scales as a package [16] and the measurement of outcomes in a large clinical sample [17] have been described elsewhere.

The structure of both the LDQ and SSQ has been investigated through application of principal component analysis (PCA). PCA is a multivariate technique applied to a set of correlated variables (or items) to identify a smaller number of uncorrelated *principal components*, which explain as much of the total variance of the original variables as possible. That way, we can say that these components provide a good representation of the original data in a more parsimonious description (a reduced number of dimensions).

PCA for the LDQ in a relatively small sample (*n* = 207) reported a single component accounting for 64.2% of the total variance [5]. Further analysis on a larger sample (*n* = 1681) yielded a first component accounting for 53.9% of the total variance [10]. The authors concluded that the LDQ measured a single construct, namely substance dependence.

In another study PCA was applied to SSQ data from 6732 addiction service users [8], yielding three reported components. All eight variables loaded highly on component I, component II related specifically to accommodation (items 1 and 2) and component III to employment and finance (items 3 and 4). The authors concluded that it did not make sense to view components II and III as distinct and thus that a single component offered the best interpretation of the data.

We have not found any studies of the dimensional structure of the CORE-10 (perhaps because it is a reduced/short form of a longer, multidimensional measure); however, the CORE Outcome Measure (CORE-OM) [6]—from which it is derived—has been extensively investigated.

Exploratory factor analysis (EFA) is another multivariate analysis technique closely related to, but distinct from, the method of PCA. EFA assumes that common causes underlie the responses given to the set of variables, or questionnaire item responses, and that these common causes lead to associations (covariance) between the items. EFA tries to identify as many common causes (‘factors’) as needed to explain this covariance as parsimoniously as possible [18].

This paper presents the results of a factor analysis to explore the relationship between the LDQ, CORE-10 and SSQ. The aim was to identify the latent factors that represent the domains measured by the three questionnaires to assess the extent to which the three scales are independent of each other, or can be understood in a smaller number of dimensions than the number of scales, without (too much) loss of information.

## Methods

A clinical sample of 715 service user records was extracted from a specialist service (Leeds Addiction Unit, UK) clinical administration database as at 2011. The LDQ, SSQ and CORE-10 were routinely administered at the start of treatment and again at one or more points between 3 and 12 months post-treatment. The three questionnaires were considered complete with up to two missing values within each scale; missing values were replaced with the mean of the completed item scores. The original dataset did not contain unique records as some service users had multiple follow-up observations. To conduct the factor analysis, unique records were extracted on the condition that they had a complete set of scores (after replacing up to two missing values) for all three questionnaires at either the first or follow-up assessment. Only one complete pre- or post-assessment record for a given service user was extracted at random.

Pairwise Spearman correlation coefficients were calculated between the three scales. Weak correlations between instruments could indicate that they constitute distinct factors. In order to determine the suitability of the data for factor analysis, the Kaiser-Meyer-Olkin Measure of Sampling Adequacy [19] and Bartlett's test of sphericity were calculated [20].

Polychoric, as opposed to Pearson, correlations were used as the univariate skewness and kurtosis values suggested variables were not normally distributed. Additionally, polychoric correlations assume only monotonic but not necessarily linear relationships between variables and they can be calculated for ordinal response scales typical on outcome measures. The method of factor extraction was unweighted least squares, with Promin rotation [21]. Oblique rotation was used as it was reasonable to assume that: (i) the instruments represent more than one factor; and (ii) the factors are correlated. Browne argued that oblique rotation is probably more appropriate in most practical situations [22].

Because previous PCAs of the LDQ and SSQ indicated a single component solution, and it seemed reasonable to assume the CORE-10 measured a distinct construct, a factor analysis extracting three factors was performed initially. Parallel Analysis (PA) [23] and Hull [24] methods were then used to determine how many factors were required to explain the variation. The PA was configured to produce 500 permuted random correlation matrices.

FACTOR 9.2 software (Rovira i Virgili University, Tarragona, Spain) was used for the factor analysis [25]. All other analyses and data preparation were conducted in stata v13 (StataCorp LP, College Station, TX, USA). Significance was assessed at the 5% level.

## Results

Of the 715 records extracted from the addiction service database, 531 related to unique individuals. The mean age was 43.4 years (range 21–78), and 56% (*n* = 296) were male (Table 1). The majority were visiting the clinic for their first episode of treatment, and nearly two-thirds were referred primarily because of alcohol use.

**Table 1:** Service user characteristicss

Characteristic		Service users (*n* = 531)
Gender, *n* (%)			*n* = 531
Male			296 (55.7)
Female			235 (44.3)
Age, years			*n* = 531
Mean (SD)			43.4 (11.6)
Median (min, max)			(21, 78)
Treatment episode number, *n* (%)			*n* = 529
1			299 (56.5)
2			126 (23.8)
3+			104 (19.7)
Referral drug, *n* (%)			*n* = 529
Alcohol			329 (62.0)
Heroin			78 (14.7)
Methadone			49 (9.2)
Other opiate			25 (4.7)
Stimulant (amphetamine, cocaine, crack)			21 (4.0)
Cannabis			12 (2.3)
Other			15 (2.8)
LDQ			*n* = 496
*Pretreatment*	Mean (SD)	15.1 (9.7)
		Median (min, max)	16 (0, 30)
*Reassessment*		*n* = 504
		Mean (SD)	6.9 (7.9)
		Median (min, max)	4 (0, 30)
SSQ			*n* = 495
*Pretreatment*	Mean (SD)	14.8 (5.5)
		Median (min, max)	15 (0, 24)
*Reassessment*		*n* = 506
		Mean (SD)	16.2 (5.4)
		Median (min, max)	17 (0, 24)
C0RE-10			*n* = 495
*Pretreatment*	Mean (SD)	20.1 (9.3)
		Median (min, max)	21 (0, 40)
*Reassessment*		*n* = 505
		Mean (SD)	14.4 (9.3)
		Median (min, max)	13 (0, 40)

CORE-10, 10-item Clinical Outcomes in Routine Evaluation; LDQ, Leeds Dependence Questionnaire; SD, standard deviation; SSQ, Social Satisfaction Questionnaire.

Outcome data were available for a higher number of patients at reassessment than at referral. On investigation, we found that this resulted from: (i) fewer completely empty questionnaires at reassessment across each instrument perhaps from patients being more engaged and willing to answer the questionnaires; and (ii) fewer missing items per questionnaire for which at least one item was completed, which made it possible to provide more complete questionnaires (see Methods section).

Higher scores for the LDQ and CORE-10 indicate a more severe dependence and level of psychological distress, respectively; however, a higher SSQ score indicates a greater sense of social satisfaction. Mean outcome scores for all three questionnaires changed favourably between assessment and reassessment (Table 1). At assessment, the SSQ was negatively correlated with the LDQ (*ρ* = –0.39, *P* < 0.001) and the CORE-10 (*ρ* = –0.47, *P* < 0.001). The LDQ and CORE-10 exhibited the strongest correlation (*ρ* = 0.65, *P* < 0.001). A small reduction in correlation between the LDQ and the SSQ was seen at reassessment (*ρ* = –0.37, *P* < 0.001) but a stronger correlation between SSQ and CORE-10 (*ρ* = –0.56, *P* < 0.001). The correlation between LDQ and CORE-10 remained virtually unchanged.

### Factor analysis

#### Dataset

A dataset with 526 mixed (pre-/post-treatment) assessment scores was used to estimate the factor structure, and a second dataset of 448 observations randomly deselected for use in the main analysis used for cross-validation of results.

As a general rule of thumb, an absolute minimum of 10 observations per variable is necessary to avoid computational difficulties in factor analysis and a sample size of greater than 500 is ‘very good’ according to Comrey and Lee [26]. The final sample size of 526 provided a ratio of over 18 cases per variable.

#### Assumption testing

The suitability of conducting a factor analysis on these data was checked using several criteria for factorability based on the covariance matrix of the variables. It was noted that every variable correlated at least 0.3 with at least one other variable. Bartlett's test of sphericity was statistically significant [χ^2^ = 9957, degrees of freedom (df) = 378, *P* < 0.001], suggesting that the 28 variables were sufficiently correlated with each other. The Kaiser-Meyer-Olkin test yielded a value of 0.95, exceeding the commonly recommended value of 0.6 [19], indicating strong partial correlations between the variables after controlling for all other variables. These tests provided evidence that factor analysis could be applied to these data.

#### Three-factor solution

Three factors accounted for 66.2% of the variance (49.2%, 10.6% and 6.4%, respectively). Each item loaded over 0.3 on only one factor, and items in the same questionnaire loaded over 0.3 on the same factor (Table 2). The LDQ items loaded on factor 1 in the range 0.75–0.99. Nine of the CORE-10 items correlated with factor 2 with loadings of at least 0.46; item 2 did not correlate above 0.3 with any factor. The eight items of the SSQ loaded on factor 3 with weights ranging from 0.52 to 0.83. The inter-factor correlations were 0.76 (factors 1 and 2), –0.47 (factors 1 and 3) and –0.56 (factors 2 and 3).

#### Two-factor solution

The Hull method advised three factors, whereas the PA method, based on 95 percentiles, recommended that two factors could be sufficient to account for the pattern of observed correlations. Also, the relatively high inter-factor correlations suggested that extracting as many as three factors may be unnecessary. The LDQ items loaded on the first factor with correlations 0.82–1.02, and the SSQ items on the second factor with weights of at least 0.63 (Table 2). Nine of the 10 CORE-10 items load above 0.41 on factor 1 (item 2 was contained within factor 2). Item 8 of the CORE-10 and SSQ items 1 and 2 had reasonable loadings on both factors; however, the high loadings of CORE-10 item 8 on factor 1 and of SSQ items 1 and 2 on factor 2 would lead us to conclude that they belong to these factors. The inter-factors correlation was –0.69.

**Table 2:** Factor loadings from the EFA

Item	Description	Three-factor solution				Two-factor solution		
		Substance dependence	Psychological distress	Social satisfaction	Communality	Psychological distress	Social well-being	Communality
LDQ 1	Preoccupation with alcohol	0.89	—	—	0.81	1.00	—	0.79
LDQ 2	Importance of drinking	0.91	—	—	0.87	0.99	—	0.83
LDQ 3	Need for alcohol	0.87	—	—	0.84	1.00	—	0.82
LDQ 4	Interference with daily activities	0.99	—	—	0.89	1.02	—	0.83
LDQ 5	Effect of alcohol	0.86	—	—	0.60	0.82	—	0.54
LDQ 6	Timing of drinking	0.92	—	—	0.79	0.94	—	0.74
LDQ 7	Desire to continue drinking	0.90	—	—	0.85	0.99	—	0.82
LDQ 8	Effect over type of drink	0.86	—	—	0.69	0.87	—	0.64
LDQ 9	Psychological addiction	0.93	—	—	0.80	0.98	—	0.76
LDQ 10	Emotional dependence	0.75	—	—	0.84	0.99	—	0.84
CORE-10 1	Nervousness	—	0.87	—	0.74	0.61	—	0.61
CORE-10 2	Support	—	—	—	0.15	—	–0.38	0.15
CORE-10 3	Coping	—	0.46	—	0.40	0.46	—	0.38
CORE-10 4	Talking	—	0.71	—	0.44	0.41	—	0.35
CORE-10 5	Terror	—	0.99	—	0.74	0.50	—	0.53
CORE-10 6	Suicidal thoughts	—	0.62	—	0.44	0.45	—	0.38
CORE-10 7	Difficulty sleeping	—	0.72	—	0.55	0.52	—	0.46
CORE-10 8	Despair	—	0.84	—	0.84	0.59	–0.33	0.74
CORE-10 9	Unhappiness	—	0.75	—	0.73	0.63	—	0.65
CORE-10 10	Distressing images	—	0.85	—	0.61	0.50	—	0.47
SSQ 1	Accommodation	—	—	0.75	0.41	0.50	0.82	0.35
SSQ 2	Living arrangements	—	—	0.83	0.55	0.38	0.90	0.48
SSQ 3	Employment	—	—	0.61	0.39	—	0.70	0.37
SSQ 4	Finances	—	—	0.61	0.42	—	0.63	0.37
SSQ 5	Social activities	—	—	0.52	0.51	—	0.67	0.51
SSQ 6	Friendships	—	—	0.60	0.51	—	0.70	0.50
SSQ 7	Relationships	—	—	0.74	0.57	—	0.82	0.52
SSQ 8	Family	—	—	0.61	0.46	—	0.73	0.46

*Note.* Factor loadings lower than absolute 0.30 are suppressed.

*Inter-factor correlations.* Three-factor solution: 0.76 (factors 1 and 2), –0.47 (factors 1 and 3) and –0.56 (factors 2 and 3); Two-factor solution: –0.6

CORE-10, 10-item Clinical Outcomes in Routine Evaluation; EFA, exploratory factor analysis; LDQ, Leeds Dependence Questionnaire; SSQ, Social Satisfaction Questionnaire.

#### Validation

The two- and three-factor models were reproduced in a different software package, Mplus (Muthén & Muthén, Los Angeles, CA, USA) [27], and the solutions obtained were structurally the same. Further information on the fit of the models to the data were provided: both described the data well [comparative fit index (CFI) and Tucker-Lewis index (TLI) >0.95] but had significant χ^2^ statistics (two factors: χ^2^ = 2144, df = 323, *P* < 0.001; three factors: χ^2^ = 1257, df = 297, *P* < 0.001). The root mean square error of approximation (RMSEA) were in the acceptable range for the three-factor model [two factors: 90% confidence interval (CI) 0.099–0.108; three factors: 90% CI 0.074–0.083]. For more information on these indices, see Schermelleh-Engel et al. [28].

As the main argument for the fit of a factor model is the replication in a different sample, a cross-validation was performed. The remaining complete cases (*n* = 448) that were randomly deselected for use in the main analysis were subjected to the same EFA. This population did not differ in demographic characteristics from the population for the main analysis. A three-factor solution accounted for 65.7% of the variance (48.6%, 11.3% and 5.8%, respectively). Communalities and factor loadings were similar to those for the three-factor model presented in Table 2. The PA method recommended two factors, as before; however, the Hull method suggested that a single factor may be sufficient to explain the variation observed in the data. Two- and three-factor models were then fitted to the validation sample using Mplus. The resulting fit statistics showed acceptable fit of both the two-factor model [χ^2^ = 785, df = 376, *P* < 0.001; root mean square error (RMSE) 90% CI 0.044–0.054; CFI = 0.985; TLI = 0.985] and the three-factor model (χ^2^ = 601, df = 375, *P* < 0.001; RMSE 90% CI 0.031–0.042; CFI = 0.992; TLI = 0.992).

## Discussion

Our aim was to examine the relationship between three outcome measures used by a UK specialist addiction service. We estimated several factor analytic models, and used two methods of dimension reduction, to assess to what extent the three scales of LDQ, SSQ and CORE-10 have common determinants (latent factors). We expected a priori that the SSQ would correlate modestly and negatively with the LDQ and the CORE-10 as measured prior to treatment and indeed this was the case. Correlation between the SSQ and LDQ has previously been reported at –0.25 in a population of addiction service users [8]. We were unable to find a study correlating the CORE-10 with the SSQ; however, in its validation, the SSQ had a reported correlation of –0.45 with the CORE-OM.

The exploratory three-factor model resulted in three correlated factors, accounting for 66.2% of the variance, which have been labelled ‘substance dependence’ (LDQ), ‘psychological distress’ (CORE-10) and ‘social satisfaction’ (SSQ). This reflects potential latent variables underpinning each questionnaire. The two-factor model accounted for 59.8% of the variance. The items from the LDQ and CORE-10 were grouped together in a factor relating to psychological distress, and the SSQ items to the factor retained from the three-factor model relating to social well-being. Although the model fit was only ‘acceptable’ for both models in the estimation sample (CFI and TLI >0.95, but RMSEA >0.05) [28], the application to the validation sample showed that the estimated factor loadings of either model were still good enough to produce useful predictions. Because this is a service-based study we used the instruments administered by this service, but others are available. In the UK there is a mandatory drug (not alcohol) reporting system that includes 20 point scales for psychological and social well-being [29]. We are not aware of mandatory measures being used elsewhere: in the USA, there was an attempt, which failed to achieve compliance, to make the Addiction Severity Index mandatory; New Zealand intends to introduce mandatory outcomes; there is an Australia version of the Treatment Outcomes Profile but as in European countries no plans for mandatory reporting. Probably the most widely used package in North America and Australasia is the Addiction Severity Index, which has been extensively evaluated and translated [30]. The Addiction Severity Index is very detailed and includes five point scales for psychological and social well-being. The scales described here are brief enough for routine use, can be embedded with other measures and they produce clinically meaningful data [17].

This study and analysis alone cannot establish conclusively whether there are two or three factors associated with addiction outcomes. Why? First, the sample is homogeneous in that it is comprised only of patients with addiction problems. While this is the relevant population in which to investigate these measures, the primary underlying problem of addiction overlays all psychological distress assessed by the instruments. To show that the CORE-10 and LDQ assess different constructs we would need a sample that contains patients who have high CORE-10 values but low LDQ values. Because this is not generally the case in this population, there was a high probability of the LDQ and CORE-10 representing a single factor. Second, there could be factors that are not captured by these three scales and could be found among outcome measures not widely used in specialist addiction services. NICE guidelines advise that assessment of adults referred to a specialist service should cover dependence, psychological and social problems, other drug misuse, physical health problems, cognitive function, and readiness and belief in ability to change [3]. These could be other factors that if assessed could constitute distinct components. Third, the conclusions drawn about the statistical dimensionality of the measures are only one aspect to consider when planning assessments in practice.

The empirical result is that the measurements obtained with the LDQ and the CORE-10 in a population with addiction problems are so closely related that it might seem hard to justify the use of both. Our interpretation of the findings is that psychological distress and social well-being are key constructs of addiction. Dependence is best thought of as a specific psychological condition subsumed under the construct psychological distress. It is clinically important because it drives substance use and is seen by clinicians as a key indicator of prognosis and the intensity of treatment an individual requires. CORE-10 on the other hand will pick up psychological distress from any source, for example, mental illness, social consequences of substance misuse and psychological adjustment post-treatment: all these should also be seen as specific conditions requiring recognition within an individual's treatment plan. Additional scales can be used to measure specific conditions—LDQ will always be central for a substance misuse population albeit it is condition specific. The items that make up the scales are a rich source of clinical material that should not be lost by simply viewing total scores.

In monitoring systems one could be more interested in following dependence more closely than general psychological distress. So a mixed assessment plan with frequent LDQ assessments, some of which are combined with CORE-10 assessments could be implemented. For those assessments where only the LDQ is assessed this information could be used to generate an estimate of the CORE-10 using regression or Item Response Methods [31] to reduce overall burden on patients and increase the speed of the individual assessments.

Limitations of this study include that findings are inferred from a relatively limited sample size from a single addiction unit. Results would have more generalisability if conducted with a larger sample from several addiction services. In addition, only particular demographic data were available from the database, namely sex and age. A larger, more strategic study could allow for the inclusion of more demographic, substance use, and physical health data that would give a clearer understanding of the population of analysis. However, for good reasons, services use a variety of scales but seldom the same package, and so such a study would present considerable challenges. The kinds of services helping people with less complex problems, which would extend the variability of the data, are not likely to collect the more comprehensive dataset needed by specialist services. Additional measures of substance use and physical health could be included in the package, but each of these present measurement challenges and the authors remain of the view that the LDQ, CORE-10 and SSQ constitute a basic set of outcomes, with a supporting evidence base, which can be used in routine practice.

## Conclusion

This analysis is the first that we are aware of to investigate the relationship between this package of well-validated measures. Statistically, we can infer that the LDQ and CORE-10 are highly correlated and may constitute a single dimension of addiction. However, clinically, this factor is difficult to label. In practice, these scales measure different, important things, namely distress/poor mental health by CORE-10 and dependence by the LDQ—the item content is very different and so both scales should be retained. Even though they correlate, our prediction is that LDQ and CORE-10 scores will change differently in long-term follow-ups and the correlation will weaken. This divergence might be important and worth detecting, which would require a longitudinal study spanning at least 2 years with multiple data collection points. In addition, the use of the CORE-10 and SSQ, as generic measures, allows for comparisons with other health areas using these same measures.
